# Massively Parallelized Pollen Tube Guidance and Mechanical Measurements on a Lab-on-a-Chip Platform

**DOI:** 10.1371/journal.pone.0168138

**Published:** 2016-12-15

**Authors:** Naveen Shamsudhin, Nino Laeubli, Huseyin Baris Atakan, Hannes Vogler, Chengzhi Hu, Walter Haeberle, Abu Sebastian, Ueli Grossniklaus, Bradley J. Nelson

**Affiliations:** 1 Multi-Scale Robotics Lab, ETH Zurich, Zurich, Switzerland; 2 Department of Plant and Microbial Biology, University of Zurich, Zurich, Switzerland; 3 Zurich-Basel Plant Science Center, Zurich, Switzerland; 4 IBM Research - Zurich, Switzerland; Texas A&M University College Station, UNITED STATES

## Abstract

Pollen tubes are used as a model in the study of plant morphogenesis, cellular differentiation, cell wall biochemistry, biomechanics, and intra- and intercellular signaling. For a “systems-understanding” of the bio-chemo-mechanics of tip-polarized growth in pollen tubes, the need for a versatile, experimental assay platform for quantitative data collection and analysis is critical. We introduce a Lab-on-a-Chip (LoC) concept for high-throughput pollen germination and pollen tube guidance for parallelized optical and mechanical measurements. The LoC localizes a large number of growing pollen tubes on a single plane of focus with unidirectional tip-growth, enabling high-resolution quantitative microscopy. This species-independent LoC platform can be integrated with micro-/nano-indentation systems, such as the cellular force microscope (CFM) or the atomic force microscope (AFM), allowing for rapid measurements of cell wall stiffness of growing tubes. As a demonstrative example, we show the growth and directional guidance of hundreds of lily (*Lilium longiflorum)* and Arabidopsis *(Arabidopsis thaliana)* pollen tubes on a single LoC microscopy slide. Combining the LoC with the CFM, we characterized the cell wall stiffness of lily pollen tubes. Using the stiffness statistics and finite-element-method (FEM)-based approaches, we computed an effective range of the linear elastic moduli of the cell wall spanning the variability space of physiological parameters including internal turgor, cell wall thickness, and tube diameter. We propose the LoC device as a versatile and high-throughput phenomics platform for plant reproductive and development biology using the pollen tube as a model.

## Introduction

Pollen tubes are one of the fastest, if not the fastest, growing cellular systems with *in vivo* growth speeds reaching around 2.7 μm/s in maize and only rivaled in the natural world by specially cultured neuronal cells [1]. The maize pollen starts to germinate within 5 minutes after contact with the stigma [2] and can grow 300 mm long in the style to fertilize the ovary, amassing along its journey a record length-diameter ratio of around 12,000. This rapid tip-growth is driven by a dynamic and precisely regulated process involving ionic exchange, cell wall material metabolism, and cytoskeletal activity [3], necessitating high-throughput-assay platforms for phenotypic quantification.

Conventional *in vitro* assays for phenotyping pollen grains and pollen tubes use multi-well plates with liquid or agar-based gel media. The spatiotemporal growth of pollen tubes is highly disordered and three-dimensional in nature with crossovers and entanglement between tubes. Furthermore, the poor adhesion of grains and pollen tubes to the substrate makes long-term quantitative analysis via high-resolution microscopy and micro-indentation difficult. The need for computer-vision assisted automation to ‘track multiple, overlapping pollen tube trajectories in fluorescent time-lapse images’ was raised at the Third Annual Pollen RCN Meeting in 2013 [4]. Real-time automation methods for micro-indentation and optical monitoring have recently been introduced [5,6], but they require costly hardware accessories to existing microscopes. Conventional *in vitro* assays lack the precise spatiotemporal control of electro-chemical stimuli in the microenvironment of the growing cells needed to study cell-cell signaling and chemo-electro tropism and guidance mechanisms, which are key to successful *in vivo* fertilization.

Microfluidics and Lab-on-a-Chip (LoC) technologies are widely used in animal cell, tissue, and organ-level research [7–9]. The crossover of these technologies into plant sciences has been limited, but is growing. Phenotyping of entire *A*. *thaliana* plants and organs, such as roots and shoots, have been demonstrated through LoC platforms like the PlantChip [10] and RootArray [6]. Pioneering work at the cellular level was reported by Palanivelu, Zohar and colleagues [11,12], where a microfluidic chip was developed to simulate the anisotropic diffusion of ovule attractants towards *A*. *thaliana* pollen tubes. The TipChip and its variants have been used to study the influence of obstacles and chemical targeting on the growth of *Camellia japonica* pollen tubes as shown by Geitmann and colleagues [13,14]. Higashiyama and coworkers have used *Torenia fournieri* to study pollen tube guidance and pollen tube-female tissue interaction and *A*. *thaliana* ovules for long-term live imaging [15,16] using specialized LoCs. All but one [15] of the above mentioned systems for pollen tubes studies lack the tight vertical confinement of the tip-growing cell in a single focal plane, which is crucial for long-term optical imaging and monitoring. The devices have a uniform height to accommodate the large size of the grain in comparison to the pollen tube, while Horade and colleagues cleverly avoided the need for a multi-height device by introducing a hand-pollinated style directly into the LoC [15]. The throughput of most existing LoC-based assays is restricted, however, as only a limited number of pollen tubes could be incorporated, guided, and observed on the chip at a time. There have been attempts at LoC-based systems for mechanical characterization of pollen tubes, but they also suffer from low-throughput [14,17] and their closed-cell architecture does not allow interfacing to calibrated micro-indentation [5], micro-gripping [18,19], micro-injection [20], or nano-indentation [21] systems for quantitative biomechanical characterization of the cell wall and cytoplasm.

Two of the most widely researched pollen tube model systems are *Lilium longiflorum* and *Arabidopsis thaliana*. Lily pollen tubes have historically been the model of choice, ever since the first electron microscopy studies of its ultrastructure [22]. Since then several physiological processes and parameters have been studied and quantified with this model, such as internal turgor pressure [23], pH and Ca^2+^ concentrations [24]. The relatively large geometric size, high *in vitro* germination rate and growth speed, and robustness of the pollen and pollen tube have been reasons for its choice as a model. With the recent release of a high quality lily pollen transcriptome [25], we believe that the use of *L*. *longiflorum* as a model will increase, requiring high-throughput analysis platforms. *A*. *thaliana* on the other hand offers the advantages of a short generation cycle, small size, and a well understood genome, transcriptome, and proteome [26–30]. With powerful forward and reverse genetic approaches, a wide mutant catalog exists for genotype-phenotype mapping. Till recently, large-scale phenotypic *in vitro* analysis of Arabidopsis pollen tubes was hindered by low pollen germination and growth rates [31,32], most likely due to lack of growth-promoting molecules found in the female pistil. Nevertheless, Arabidopsis pollen remains the most studied model for pollen tube growth and its regulation.

In this paper, we report the concept of a species-independent LoC platform for long-term, high-resolution optical observation and mechanical measurements of pollen tubes. We show devices specifically tailored to study *L*. *longiflorum* and *A*. *thaliana* pollen tubes. We have demonstrated the unidirectional growth of hundreds of lily and Arabidopsis pollen tubes with no significant changes in growth parameters such as morphology, germination, and growth rates as compared to conventional *in vitro* plate culture. We demonstrate the integration of the LoC device with the CFM [5,33,34] to characterize the cell wall stiffness of lily pollen tubes. The high-throughput mechanical measurements of the LoC-CFM combination in conjunction with FEM modeling allowed us to determine the uncertainty estimates of the linear elastic moduli of the lily pollen tube cell wall. We believe that this LoC platform will significantly aid bio-chemo-mechanical phenotyping as well as systems-modeling of the mechanisms governing pollen tube growth.

## Materials and Methods

### Lab-on-a-Chip device fabrication

The photolithography mask is designed using Siemens NX CAD software and printed in film by Selba A.G, Switzerland. From the photomask to the final LoC device, the process entails a two-step photolithography followed by PDMS casting, cutting, and glass bonding (see Fig A in S1 File). In the first step, commercial photo-curable polymer SU8 (Microchem Corp, U.S.A) is spin-cast onto a 4 inch silicon wafer to reach the desired micro-channel height. After soft-baking on a hot-plate, the wafer is exposed to UV light with the first layer mask to generate the channels. After a post-exposure baking, the unpolymerized resist is washed off and baking is done once again to make the mold mechanically stable and adherent to the silicon substrate. A second layer of SU8 is spun-cast to the height required for the grain chamber, and the baking, exposure, resist removal, and baking steps are repeated to generate the two-height SU8 mold. Then PDMS is poured into the mold under a vacuum pump and baked at 80°C to crosslink the PDMS. After cooling, the PDMS can be peeled off and then cut into the required size. A 1.5 mm biopsy punch needle is used to punch the fluid inlet holes and the PDMS chip is cleaned with tape. An oxygen plasma chamber is then used to bond the PDMS to a microscopy slide or coverslip. To improve adhesion of the pollen tubes to the device after they grow out of the microchannels of the LoC, the glass slide is coated with poly-L-lysine.

### Plant material

Lily (*Lilium longiflorum*) flowers are purchased from the local florist in Zurich and the anthers excised and individually placed in Eppendorf tubes and kept at -80°C for storage. On the day of experimentation, the Eppendorf tube with the anther is allowed to equilibrate to room temperature for 30 minutes at 100% humidity. The culture medium for pollen germination consists of: 160 μM H_3_BO_3_, 130 μM Ca(NO_3_)_2_, 1 mM KNO_3_, 5 mM MES, 10% sucrose at a pH of 5.6. For conventional *in vitro* studies, the pollen is brushed onto microscopy slides and then covered with a few drops of growth medium. For in-chip studies, the culture medium is added into the anther-containing Eppendorf tube, and the system is allowed to imbibe for thirty minutes before the mixture of culture media and pollen grains are taken up into a syringe for loading into the LoC device.

Arabidopsis [*Arabidopsis thaliana* (L.) Heynh., accession Columbia (Col-0)] plants are grown under controlled long-day conditions at 22°C and 60% relative humidity. Dehiscent flowers are harvested and kept at 100% relative humidity for pollen rehydration for about 30 minutes. To collect the pollen grains, approximately 30 flowers are immersed in a 2 ml Eppendorf tube containing 1.5 ml of pollen germination medium (1.6 mM H_3_BO_3_, 5 mM CaCl_2_, 5 mM KCl, 1 mM MgSO_4_, 10% sucrose, pH 7.5). Flowers are slightly squeezed with tweezers, then briefly vortexed. After centrifugation at 950 rcf for 2 minutes, floral tissue is removed with tweezers. The pollen grains are pre-incubated at 22°C in the Eppendorf tube for 30–60 minutes before loading into the LoC device.

### Germination and growth in LoC device

After pre-incubation in the growth medium the grains are injected into the LoC inlet with light pressure using a microsyringe. The loading pressure flushes the grains from the inlet into the grain reservoir, while the liquid medium flushes in through the microchannels, which are open at the end. Each unit cell is individually filled with the grain/growth medium mixture. A droplet of growth medium is placed on top of each inlet, and the LoC is placed in a humid environment and under controlled temperature. The first pollen tubes germinate and enter the channel within an hour of incubation.

### Micro-Indentation with the Cellular Force Microscope

The hardware of the micro-indentation system is identical to that described by Felekis and colleagues [5]. The targeted growing pollen tube is located with the inverted microscope. The sensor tip is positioned as close as possible to the pollen tube. First, a coarse approach is performed with the coarse positioners to find the location of the glass surface. This approach step is controlled to approximately 500–600 nm/sec and, after the contact to the glass slide the sensor tip is lifted up by 70 μm. The tip is then positioned over the tube and a fine approach and micro-indentation is performed using piezo-positioners. A maximal loading force of 5 μN and a loading/unloading speed of 2 μm/sec is used across the experiments. The measurements are done with the LoC, the sensor tip and the pollen tube completely immersed in the growth medium. There is no active fluid flow and the large fluidic volume around the LoC ensures that there is minimal evaporation during the course of measurements. The capillary stiffness experienced by the sensor tip is two orders of magnitude lower than the stiffness of the tube cell wall and is thus neglected. The force-indentation data is processed in MATLAB and the sensor stiffness is cancelled from each dataset to yield the true force-indentation curve (see Fig B in S1 File). An image is captured with the inverted microscope immediately after every indentation to determine the position of the indenter with respect to the growing tube. The apparent stiffness defined as the slope in the region of maximum load is calculated for the loading and unloading curve separately to account for the viscoelastic behavior of the pollen tube cell wall. For the FEM modeling, we only used the loading curve dataset.

## Results and Discussion

### Design and fabrication of the Lab-on-a-Chip device

**Fig 1:**
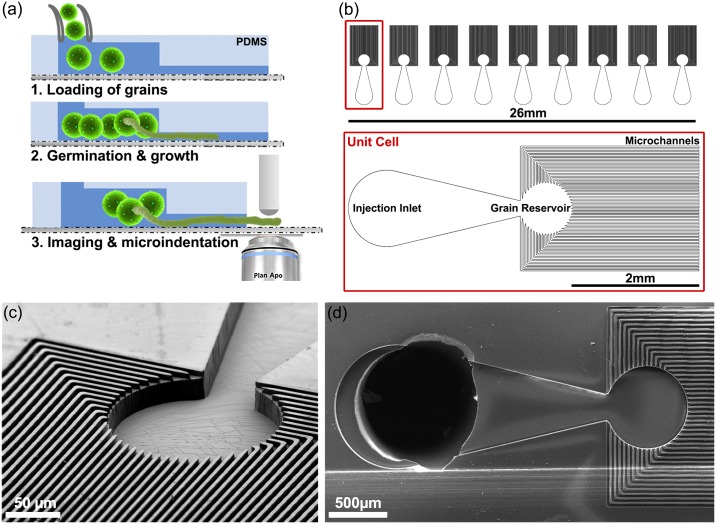
Lab-on-a-chip (LoC) device. (a) Design concept and functionality—The LoC is loaded with grains and nutrient medium, the grains germinate, and the pollen tubes are self-guided into the microchannels, allowing for massively parallelized optical imaging and micro-indentation. (b) The design layout of the lily LoC with a magnified view of an individual cell. (c),(d) Scanning electron micrographs of the fabricated PDMS chip for lily.

The LoC design, demonstrated here for *L*. *longiflorum* (Fig 1), is a species-independent platform allowing for optical observation via bright field, differential interference contrast (DIC), or fluorescent-confocal microscopy of massively parallelized unidirectional growth of pollen tubes in the same focal plane. The vertical confinement of the tubes eliminates the need for constant objective refocusing for long-term microscopy and the single directional and parallelized guidance allows for easier automation and post-processing of growth rates and other morphological assessment. Furthermore, the open channel architecture of the chip (Fig 1a) enables interfacing with well-established experimental platforms, such as the CFM and AFM for mechanical and surface morphology characterization of the cell wall [35,36], or to micro-injection systems for intra-cellular injection of dyes or internal turgor pressure measurement [23], as well as for chemical, electrical, thermal, or osmotic modification of the micro- or macro-environment around the growing pollen tube [37–40]. The design easily allows for fluorescent dye loading via passive diffusion after germination [41] and by pressure shock in non-germinated pollen [42].

The basic functional unit of the LoC, called the unit cell (Fig 1b), consists of three sections: (i) a large circular fluidic chamber that serves as the inlet for loading a pollen-growth medium, (ii) a grain reservoir connected to the inlet via a tapering neck, and (iii) dozens of collinear microchannels emerging from the grain reservoir. Guided by the injection fluid pressure, the grains flow through the inlet into the grain reservoir where they can germinate. The surrounding channels guide the pollen tubes during growth and parallelize their growth in a unidirectional trajectory in the same focal plane. Our two-height microfluidic chip is fabricated via two-step photo-lithography, followed by soft-replica molding of polydimethylsiloxane (PDMS), which allows for a higher height for the pollen grain chambers with the tubes growing in a narrower channel (Fig 1c). The choice of PDMS for the device is due to its optical transparency, low autofluorescence, high permeability to oxygen and carbon dioxide, low cost, and ease of device fabrication.

Each lily LoC occupies a 26x10 mm space with nine identical unit cells placed next to each other (Fig 1b). In comparison, a standard microscopy slide has dimensions of 75x26 mm. Each unit cell has 44 microchannels emerging from the grain chamber allowing for a theoretical maximum of 9x44 = 396 tubes to be simultaneously and unidirectionaly guided. Considering that the lily pollen tube must grow up to 120 mm through the style to fertilize the female gametophyte, and pollen growth rates of 100–500 nm/sec have been recorded, we designed the chip such that the shortest microchannel is 2 mm in length and the longest 3.4 mm, which allows for the observation of several hours of growth.

From a design and fabrication point of view, there is no technical limitation on the maximum channel length that can be made by this process. Shorter channel lengths allow for reduced experimental time in micro-indentation studies, as the pollen tubes grow out of the microchannels onto the glass slide quicker. This can be easily achieved by shortening the PDMS channel length by a blade-cut. With the non-uniform length distribution of the channels, the traversal length for each potentially guided pollen tube is different, allowing for sequential micro-indentation as they emerge out of the channels. To tailor the exact dimensions for the lily and Arabidopsis chip variants, we assumed the geometry of the pollen grains to be well approximated by a prolate ellipsoid and the pollen tube by a right-circular cylinder. We measured a major diameter of 128.5±9.9 μm and minor diameter of 98.3±5.8 μm for lily pollen (n = 40) and, correspondingly, 27.0±1.8 μm and 19.9±1.1 μm for Arabidopsis pollen (n = 40). The tube diameters are 17.4±2.5 μm and 4.9±0.7 μm for lily and Arabidopsis (n = 40), respectively. For the lily LoC, the design width and height of the channels are chosen to be 25 μm and 30 μm, respectively, allowing for adequate flow of nutrients and non-constricted growth of the pollen tube in the channel. We achieved a width of 24.9±0.7 μm and height of 31.9±0.7 μm as confirmed by the analysis of SEM images (Fig 1c and 1d). The depth of the inlet region and the grain reservoir is 118.5±9 μm (design value of 120 μm), allowing the flow of grains without multilayering or stacking.

### Germination, growth and parallel guidance

**Fig 2:**
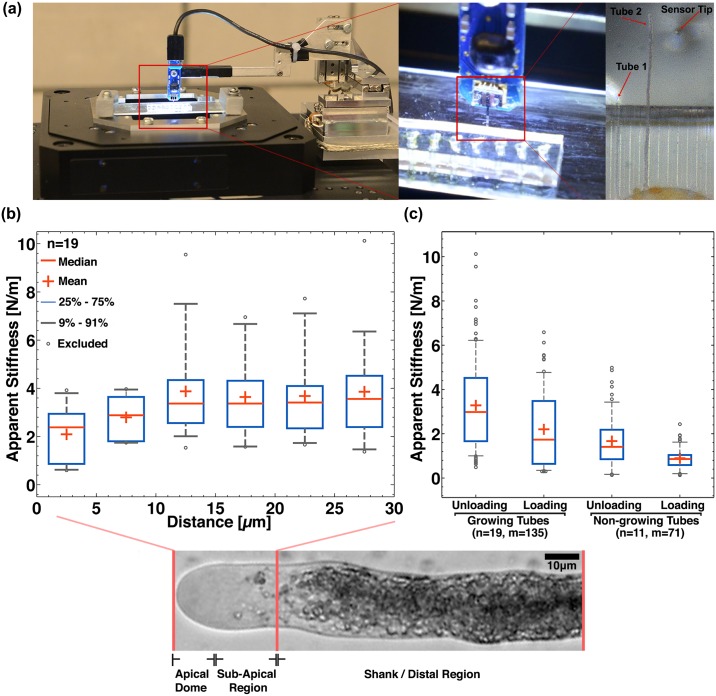
Germination, growth, and parallel guidance of pollen tubes in the LoC device. (a) The LoC is injected with nutrient medium containing lily pollen grains that become concentrated and appear as yellowish circles. (b) A view of a lily unit cell immediately after injection of grains. (c) Three lily pollen tubes are guided into neighboring channels and can be simultaneously imaged at high magnification. (d) A stitch of the three unit cells shows the equifocal unidirectional guidance of a large number of lily pollen tubes. (e) This stitch shows the guidance of eleven *A*. *thaliana* pollen tubes in a single unit cell. N = number of tubes guided in a unit cell.

The germination rate of lily grains seems to be unaffected in the LoC and the tube morphology looked similar to those of control tubes grown on liquid medium-based slide assays. We recorded an average of 12 pollen tubes guided per unit cell (n = 34) 7 hours after chip-loading (Fig 2a, 2b, 2c and 2d), yielding an equi-focal unidirectional guidance of 9x12 = 108 lily tubes per LoC device. Such high rates of parallelized, directional tube growth without entanglement, allows quantitative phenotyping at unprecedented rates. Moreover, there is no change in diameter of the pollen tubes in the chip (17.31±2.4 μm, n = 18) compared to control tube diameters. The lily tubes showed regular oscillatory tip growth [43] (see Fig C in S1 File) and the average *in chip* growth rate is 189 nm/sec (n = 14) compared to an *in vitro* control rate of 272 nm/sec (n = 14). The viability of the tubes is not affected as *in vitro* growth rates with a high variability of 100–500 nm/sec have been reported in the literature [39,44]. *Torenia fournieri* pollen tubes grown in microchannels have been reported to show a 2.5 times enhanced growth rate compared to normal liquid medium assay (n = 16), and it was speculated that the microchannels mimic an *in vivo* growth environment for the pollen tubes [15]. In the TipChip [13], it was observed that changing the microchannel-height to tube-diameter ratio from 4.7 to 9.4 increased the growth rate by up to 50% (n = 3) for *Camellia japonica*, but no control data on conventional *in vitro* growth rates was presented. We also achieved a large guidance rate for Arabidopsis pollen tubes in the LoC device (Fig 2e). The average number of tubes guided per unit cell was 6 (n = 24 cells). The Arabidopsis LoC design dimension accommodates 40 unit cells because of the smaller grain and tube size as compared to lily. With the increase in unit cells per LoC, we can thus uni-directionally guide on average 6x40 = 240 Arabidopsis tubes per chip.

No tube growth inhibition due to the L-shaped bends of the channel is observed. The tubes successfully navigated the bends without a change in growth rate. Even in sharp bends no tip bursting is observed (Fig 2c) and the pollen tubes are able to grow through the entire length of the channels and exit the PDMS device onto the glass slide. After exiting the channels, the tubes grow in a straight direction for several hundred micrometers before changing to a random growth direction. This single-directional growth is important for robust localization and automation of single cell mechanical indentation studies. The growth in the microchannels is reminiscent of *in vivo* conditions in the stylar matrix, which force individual pollen tubes to navigate maze-like trajectories to reach the female gametophyte. After germination, lily pollen tubes traverse through a hollow pistilar environment, adhering to the epidermal cells aligning the transmitting channel while, in Arabidopsis, the pollen tubes penetrate the cell wall of stigmatic papillar cells and grow intercellularly through the transmitting tissue to reach the ovules [45].

To demonstrate the low auto-fluorescence and compatibility of the LoC device with fluorescence microscopy, we labeled the cell wall using propidium iodide (PI) and monitored intracellular calcium concentration using the dye Calcium Green^™^-1 AM (see Fig D in S1 File). While lily pollen tubes can be loaded with fluorescent dyes via particle bombardment, electroporation, or—less invasively—by osmotic pressure [42], we chose to use the cell wall permeable AM-ester form of Calcium Green (albeit its low sub-cellular resolution and sequestering into vesicles) and PI because it can be co-incubated with the tubes in the growth medium.

### Integration with Cellular Force Microscope for high-throughput micro-indentation

**Fig 3:**
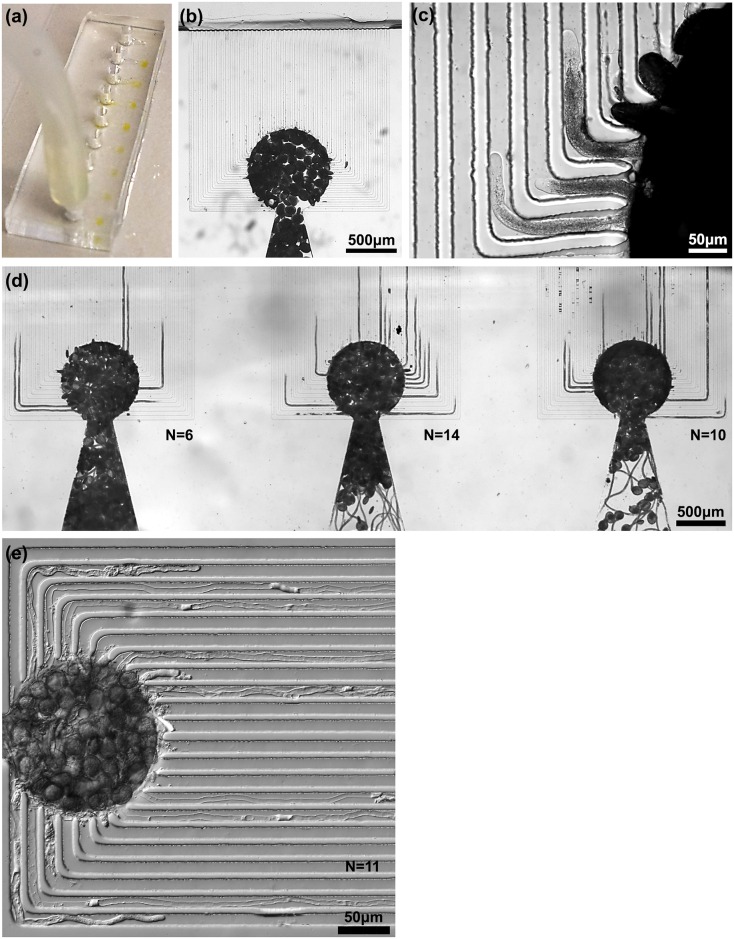
System integration of the LoC with the Cellular Force Microscope and micro-indentation dataset. (a) High-throughput micro-indentation measurements are possible because directionally guided tubes emerge out of the channels. (b) The apparent stiffness (unloading) of growing tubes is measured along the length of the tube near the apex region. (c) The apparent stiffness (loading and unloading) of the shank area of growing lily tubes compared to that of non-growing tubes. (n denotes the number of tubes and m denotes the total number of indentations on n tubes).

The cell wall is a heterogeneous structural network of polysaccharides and proteins, and the understanding of its bio-chemo-mechanics is of utmost scientific, agricultural, and socioeconomic interest, arising from the use of plant cell wall material for food, feed, fiber, fuel, paper, wood, adhesives, coatings, and thickeners [46]. The regulation of the spatiotemporal rheology of the cell wall is crucial for cellular differentiation and morphogenesis, as well as for mechanical stability and restraint against pathogens and environmental factors like wind, rain, and composition of the ground. Complementary to organism and tissue level studies on mechanical aspects of growth and morphogenesis, pollen tubes are an ideal *in vitro* system for studying biomechanics at a cellular level. Previously reported micro-indentation studies on pollen tubes suffered from low measurement throughput and have mostly used micron-sized indenter geometries—*Papaver rhoeas* (pollen tube diameter, d_t_ < 10 μm and indenter tip diameter, Φ = 3–4 μm [47]), *Solanum chacoense* (d_t_ < 10 μm Φ = 10 μm [48]), *A*. *thaliana* (d_t_ ~ 5 μm Φ = 3–4 μm [49]) and *L*. *longiflorum* (14 < d_t_ < 20 μm Φ = 4 μm [50] and Φ = 0.8 μm [51]). A high-throughput micro-mechanical characterization system is achieved by integrating the LoC device with the well-established CFM platform (Fig 3a). After germination and guidance, when the first tubes begin to emerge from the microchannels, rapid micro-indentation is performed on the tubes within the first 200 μm of their growth outside the channel with a sub-micron tipped-indenter (tip diameter, Φ = 800 nm, see Fig E in S1 File). The microchannel guidance enables a predictable and uni-directional growth of the tubes out of the PDMS chip onto the poly-L-lysine coated glass slide (Fig 3a). This ensures increased cellular localization and adhesion for performing rapid micro-indentation. The slope of the measured force-indentation curve is defined here as the *apparent stiffness*, since the curve does not solely represent the mechanical behavior of the cell wall, but also the contribution of the cell and indenter geometry, along with the cell’s turgor pressure. In general, the curves exhibit mild hysteresis or viscoelastic behavior (see Fig B in S1 File) and, hence, we calculated the loading and unloading apparent stiffness separately.

We perform micro-indentation along the length of growing lily pollen tubes (n = 19). The force-indentation curves reveal a reduction in the measured apparent stiffness at the apex of the pollen tube compared to the distal region as shown in Fig 3b. We believe that the measurement of reduced apical stiffness is the result of at least two effects, the change in contact-geometry between probe and pollen tube cell wall and also the gradient in the biochemical constituency of the cell wall along the length of the pollen tube [51,52]. The contact-angle of micro-indentation at the apical dome is less than 90°, leading to a reduction in the reaction force acting along the force-sensor axis. Secondly, cell wall staining shows a gradient in molecular composition across the length of the lily pollen tube [53]. The apical dome is rich in methyl-esterified pectins and non-crystalline cellulose compared to the distal region. De-esterified pectins are absent in the apical dome and are found uniformly across the shank area. Lastly, the presence of callose steadily increases from the apex to the distal region and crystalline cellulose is present in uniform intensity across the whole length of the tube. A combination of the gelatinous nature of the methyl-esterified pectin concentrated at the apex and the geometric effect of a non-normal contact indentation can lead to the reduced stiffness measurements at the apex. With conventional single-axis micro-indentation methods, it is difficult to differentiate between these two effects, and multi-degree of freedom force sensors [54,55] or AFM-based nano-indentation techniques are needed to determine the contribution of the geometry and biochemical composition effects.

We compared the apparent stiffness of the distal or shank region (50 μm away from the tip) of growing lily pollen tubes to that of non-growing pollen tubes. Untriggered or natural growth-arrest, a state of negligible tube growth but displaying active internal streaming, is commonly observed in *in vitro* assays. The distal stiffness measured on growing pollen tubes (n = 19, 135 indentations) can be characterized by the mean and median loading (unloading) stiffness of 2.20 N/m (3.28 N/m) and 1.73 N/m (2.98 N/m), respectively (Fig 3c). The broad stiffness distribution is attributed to intra-cellular, inter-pollen, inter-anther, and inter-flower variability as the micro-indentation technique, in itself, is robust and repeatable. Compared to growing tubes, indentation of non-growing pollen tubes (n = 11, 71 indentations) reveal a significant reduction in the mean loading (unloading) stiffness 0.69 N/m (1.67 N/m).

Naturally growth-arrested *Papaver rhoeas* tubes were previously reported to exhibit lower distal stiffness compared to growing tubes and this was posited to be due to reduced turgor pressure [56]. While it is well known from osmotic assays that a minimum level of turgor is necessary for pollen tube growth, no correlation was observed between internal turgor levels, measured and manipulated with a micropipette, and the growth rate in lily tubes [23]. While there has been no other direct measurements of turgor pressure in pollen tubes, micropipette-based techniques used on geometrically isotropic *Chara corallina* (green algae) cells have reported a linear correlation between growth and internal turgor [57]. The extension response of tip-growing fungal hyphae to changes in internal turgor show a more complicated relationship [58], with even reports of normal tip-growth in *S*. *ferax* taking place in the absence of any measurable turgor pressure, achieved through softening of the cell wall in the region of growth [59]. While an internal state of reduced turgor is a possibility, further micro-mechanical investigations using advanced tools like the fluidic AFM [60] could provide a means of simultaneous turgor manipulation and force-indentation on pollen tubes.

A comparative study of the mechanical properties of the cell wall across pollen species and measurement techniques based on apparent stiffness data is difficult because the measurements are specific to the particular indenter geometry used, the tube diameter, internal turgor pressure, cell wall thickness, and the biochemistry-induced mechanical anisotropy of its cell wall. Assuming a linear elastic material behavior of the cell wall combined with knowledge about cell wall thickness and internal turgor, one can estimate the effective Young’s modulus for the entire cell wall using FEM-based modeling, taking into account the known geometrical parameters of the micro-indentation. The FEM-based modeling framework presented by Vogler and colleagues [51] is implemented and the effect of the biologically relevant variability in turgor, cell wall thickness, cell diameter, and the Young’s modulus on the apparent stiffness during micro-indenter loading is investigated. We observe that several different combinations of these input parameters, and especially an order of magnitude spread in Young’s moduli from 20 MPa to 400 MPa, yield similar values of loading stiffness (see Table A and Fig F in S1 File). This is in the range of recently published measures of elastic moduli of plant cell walls, 20 and 90 MPa for lily pollen tubes [51], 280–420 MPa for *Camellia japonica* pollen tubes [17], and 50–757 MPa for *Nicotiana tabacum* Bright Yellow-2 (BY-2) cells [61]. Whole cell compression tests have been used to estimate the cell wall moduli of *Saccharomyces cerevisiae* to be between 107–112 MPa, which are fairly consistent within the various phases of growth [62]. The cell wall stiffness of fungal hyphae was quantified to be between 64–110 MPa using quasi-static AFM [63].

These micro-indentation studies show that we need a statistical approach to quantify the mechanics of the pollen tube cell wall. One should refrain from using a single value attribution to either the apparent stiffness or the effective linear elastic moduli of the pollen tube cell wall. A key reason is that the cell wall is a heterogenous polymer with spatiotemporal modulation of its underlying biochemistry. It is also due to the currently unobservable dynamic nature of cell wall thickness and turgor pressure, which vary depending on the growth environment *in vivo* or *in vitro*. Importantly we must note that the estimate of the Young’s modulus is highly dependent on the underlying modeling approach used, and this explains the discrepancies between the estimates in literature, which have utilized different modeling approaches. Quantified measurements of turgor pressure, effective cellular stiffness and a consistent modeling paradigm to determine the cell wall elastic moduli need to be established, if we are to unravel the mechanisms underlying pollen tube growth and penetration through the stylar matrix.

## Conclusions

We designed and introduced a LoC device for germination, growth, and unidirectional guidance of hundreds of pollen tubes in the same focal plane. The two chip designs demonstrated in this paper for lily and Arabidopsis can be directly used for other well-studied pollen tube models. The lily chip with its 25 μm wide channels can be used to guide *Camellia japonica* (camellia), *Nicotiana tabacum* (tobacco), and *Zea mays* (maize) pollen tubes, while the Arabidopsis design can be used for *Papaver rhoeas* (popy) *and Solanum chacoense* (wild potato) pollen tubes and fungal hyphae. Early adoption of these cost-effective LoC devices by the community can aid development of optimized in-chip germination and growth protocols for different wild type species and their mutant lines. The LoC devices are fully compatible with calibrated and robust micro-mechanical characterization platforms like the CFM, which ensure repeatability across studies on growth biomechanics. We used this integrated LoC-CFM platform for biomechanical characterization of growing and non-growing lily pollen tubes. Using the micro-indentation dataset, the uncertainty estimates in the physiological growth parameters and FEM modeling, we have shown that there exists a large range in the effective linear elastic moduli of the lily pollen tube cell wall. We believe that our LoC can serve the need for high-throughput, long-term live cell imaging and micro-mechanical characterization towards unraveling the causality chain between the oscillatory growth variables of ion fluxes, localized exocytosis, cell wall remodeling, turgor pressure, and growth rates generating the fast tip-polarized cell growth in pollen tubes and fungal hyphae.

## Supporting Information

S1 File Contains supporting figures and the parametric finite element (FE) model and associated results. (DOCX)
