# Correction: Massively Parallelized Pollen Tube Guidance and Mechanical Measurements on a Lab-on-a-Chip Platform

**DOI:** 10.1371/journal.pone.0171981

**Published:** 2017-02-08

**Authors:** Naveen Shamsudhin, Nino Laeubli, Huseyin Baris Atakan, Hannes Vogler, Chengzhi Hu, Walter Haeberle, Abu Sebastian, Ueli Grossniklaus, Bradley J. Nelson

The images for Figs 2 and 3 are incorrectly switched. The image that appears as Fig 2 should be Fig 3, and the image that appears as Fig 3 should be Fig 2. The figure captions appear in the correct order.

**Fig 2:**
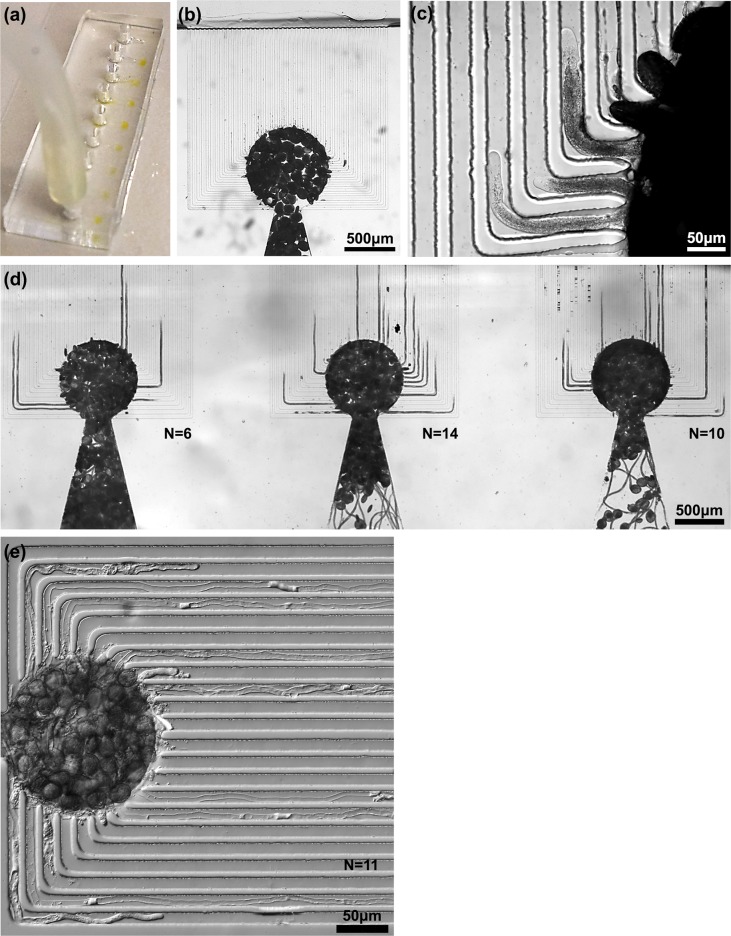
Germination, growth, and parallel guidance of pollen tubes in the LoC device. (a) The LoC is injected with nutrient medium containing lily pollen grains that become concentrated and appear as yellowish circles. (b) A view of a lily unit cell immediately after injection of grains. (c) Three lily pollen tubes are guided into neighboring channels and can be simultaneously imaged at high magnification. (d) A stitch of the three unit cells shows the equifocal unidirectional guidance of a large number of lily pollen tubes. (e) This stitch shows the guidance of eleven *A*. *thaliana* pollen tubes in a single unit cell. N = number of tubes guided in a unit cell.

**Fig 3:**
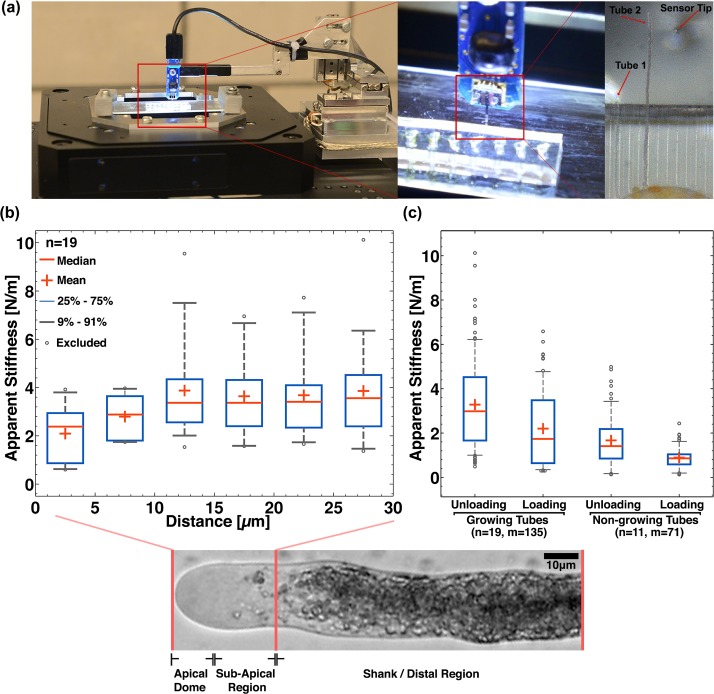
System integration of the LoC with the Cellular Force Microscope and micro-indentation dataset. (a) High-throughput micro-indentation measurements are possible because directionally guided tubes emerge out of the channels. (b) The apparent stiffness (unloading) of growing tubes is measured along the length of the tube near the apex region. (c) The apparent stiffness (loading and unloading) of the shank area of growing lily tubes compared to that of non-growing tubes. (n denotes the number of tubes and m denotes the total number of indentations on n tubes).
