# Supplementary material for: Massively Parallelized Pollen Tube Guidance and Mechanical Measurements on a Lab-on-a-Chip Platform
Source: PLoS One. 2016 Dec 15;11(12):e0168138. doi: 10.1371/journal.pone.0168138 (PMC5158026; doi:10.1371/journal.pone.0168138)
Supplement: S1 File — (DOCX) [file pone.0168138.s001.docx]

**S1 Appendix: Supporting Information**


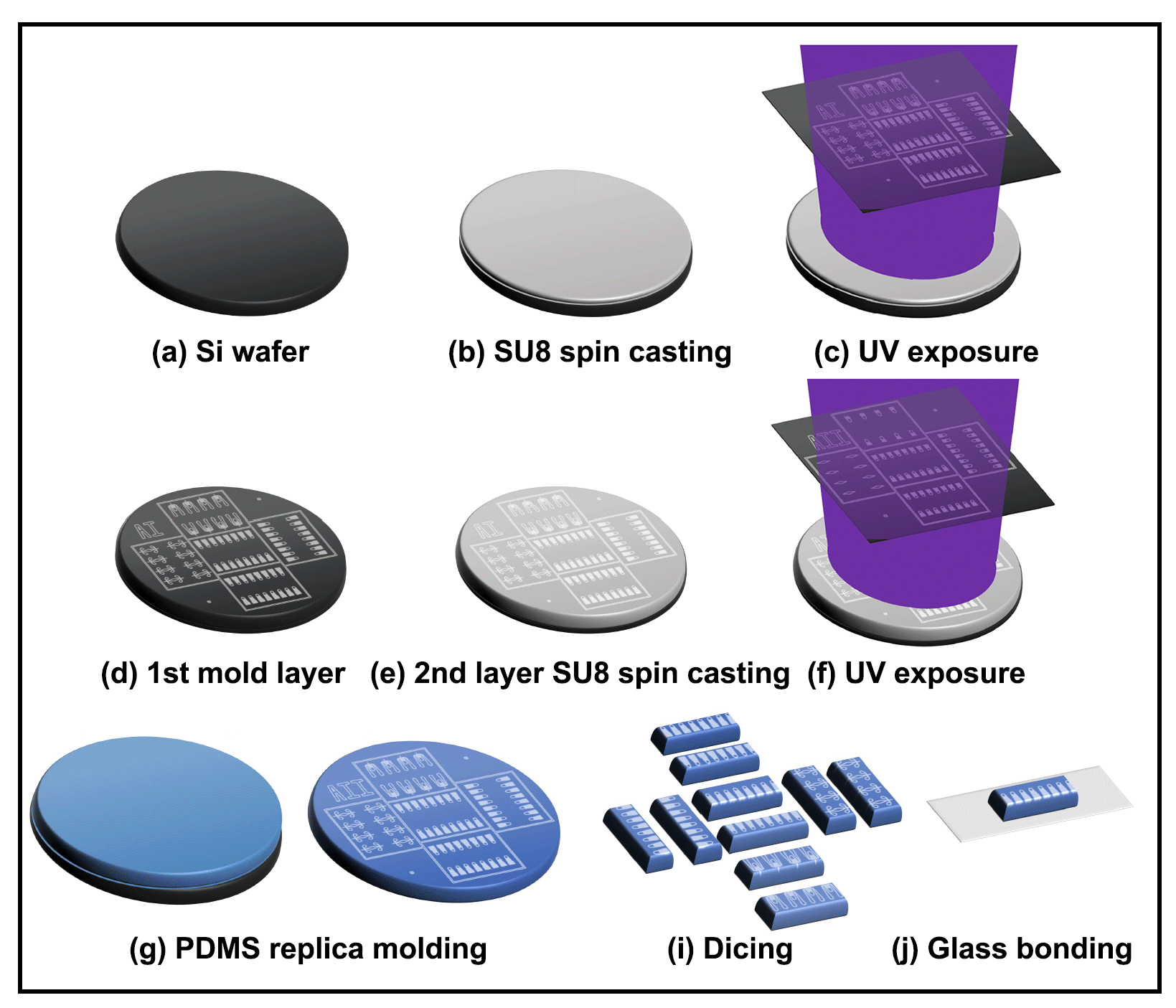


Fig A. Fabrication process of the Lab-on-a-Chip device.

Fig B. An example of a force-indentation curve on lily pollen tube.

Fig C. Oscillatory tip growth of a lily pollen tube in the chip.


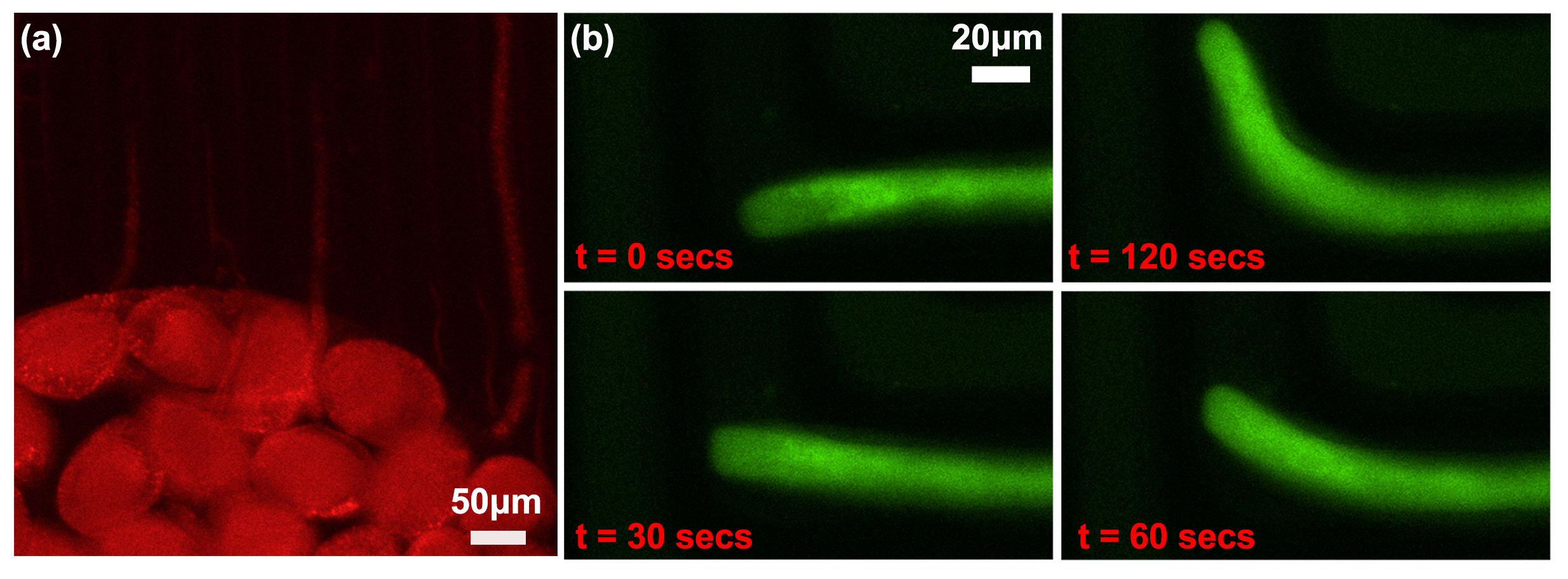


Fig D. Passive co-incubation staining of lily grains and tubes (a) Cell wall staining with propidium iodide and (b) Calcium Green^TM^-1 AM stained tube navigates a 90 degree bend in the microchannel.

**
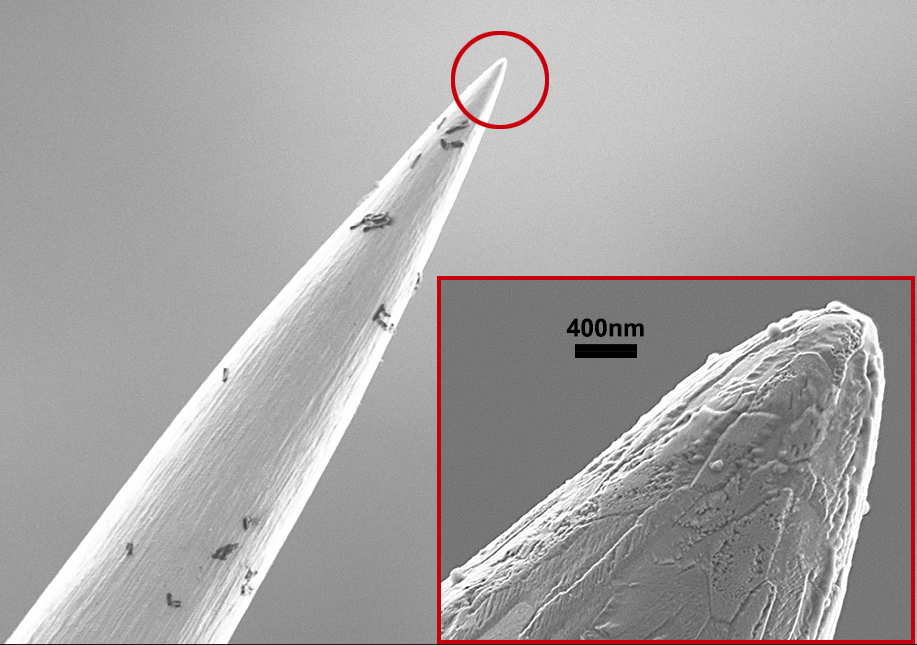
**

Fig E. SEM of CFM micro-indentation tip (Tungsten). The nominal tip diameter is 0.8 μm.

**Finite Element Method (FEM)-based Modeling**

**Model Implementation**

The mechanical modeling of the micro-indentation is implemented in COMSOL Multiphysics® Structural Mechanics module. In the three-dimensional FEM simulation environment, the pollen tube is geometrically modeled as a long cylindrical shell capped with a hemispherical dome and uniformly pressurized from within. A linear orthotropic material model is used for the cell wall, with the formulation as detailed previously [1,2]. Furthermore, the cell wall is assumed to be uniform in thickness and fully compressible, with the same radial and longitudinal elastic moduli. The shear modulus equals half of the longitudinal elastic modulus and the turgor pressure is homogenous across the whole volume. A mean circumferential stretch ratio $\lambda_{c}$ =10.2 % is obtained by pollen tube plasmolysis (n=16), i.e., by replacing the growth medium with 15 % mannitol solution. The longitudinal stretch ratio $\lambda_{l}$is assumed to be half the circumferential stretch ratio [1]. The simulation is solved in a two-step process, where the non-turgid tube is pressurized with a given turgor pressure and in the second step the indenter is brought in contact with the tube and the indentation progresses iteratively.

We performed a parametric FEM study to account for the variability in turgor pressure, cell wall thickness, the overall dimensions of the pollen tube, and the elastic moduli on the experimentally measured spread in loading stiffness of the pollen tube. The indentation problem is solved for a multi-parameter range of cell wall thickness (100-700 nm), tube diameter (17.3±2.3 μm) and turgor pressure (0.21, 0.30, and 0.4 MPa) reported for lily pollen tubes. We use a logarithmic strain model combined with the thin-walled pressurized cylinder approximation [3] to estimate the range of circumferential and longitudinal elastic moduli ($E_{c}$and $E_{l}$) and the initial unpressurized pollen tube diameter for our simulations,

$$E_{c}=\frac{Pr}{tlog( \lambda_{c})}; E_{l}=\frac{Pr}{2t\log(\lambda_{l})}$$

where $P$ is the turgor pressure, $r$ is the radius of tube, and$t$ is the cell wall thickness. The linear elastic moduli thus become dependent variables and have been summarized in Table S3 for the range of cell wall thickness and turgor pressure used in combination with the measured range of tube diameters. The indentation point is always 60 μm away from the apex of the tube.

**Choice of Input Parameters**

Even though the ultrastructure and physiology of lily pollen tubes have been studied since the 1960s, there is a broad range in the measured values of cell wall thickness and turgor pressure, which are key parameters needed to estimate the elastic moduli. Cell wall thickness has a spatiotemporal modulation across the tube. It is thicker at the apex than in the shank, signifying the conversion of methyl-esterified pectins into Ca^2+^-mediated cross-linked structures further down the length [4]. In addition, the apical cell wall in lily has been shown to modulate in thickness from 500 nm to 700 nm [5], with a period of 26 seconds corresponding to the growth oscillation cycle. From transmission electron microscopy (TEM) images, the reported thickness has a range from 110 nm to 400 nm [6], while an upper bound of 700 nm was previously reported using propidium iodide to stain the cell wall [1]. A measurement of the turgor pressure of lily pollen has been reported only twice. A value of 0.209±0.064 MPa (n=106) was reported for growing lily tubes using micro-pipette injection and a value of 0.79 MPa (n=49) by incipient plasmolysis [7], while an internal pressure of 0.317±0.07 MPa (n=17) was reported in lily pollen grains by Pertl et al. [8], who also used a micro-injection technique.

| **Turgor pressure (MPa)** | **Cell wall thickness (nm)** | | | | | | | **Tube diameter (μm)** |
| --- | --- | --- | --- | --- | --- | --- | --- | --- |
|  | **100** | **200** | **300** | **400** | **500** | **600** | **700** |  |
|  | **Elastic moduli (MPa)** | | | | | | |  |
| **0.21** | 161.41  165.25 | 80.70  82.63 | 53.80  55.08 | 40.35  41.31 | 32.28  33.05 | 26.90  27.54 | 23.06  23.61 | **15** |
| **0.21** | 185.08  189.49 | 92.54  94.74 | 61.69  63.16 | 46.27  47.37 | 37.02  37.90 | 30.85  31.58 | 26.44  27.07 | **17.3** |
| **0.21** | 210.90  215.93 | 105.45  107.96 | 70.30  71.98 | 52.73  53.98 | 42.18  43.19 | 35.15  35.98 | 30.13  30.85 | **19.6** |
| **0.30** | 230.58  236.07 | 115.29  118.04 | 76.86  78.69 | 57.64  59.02 | 46.12  47.21 | 38.43  39.35 | 32.94  33.72 | **15** |
| **0.30** | 264.39  270.69 | 132.19  135.35 | 88.13  90.23 | 66.10  67.67 | 52.88  54.14 | 44.07  45.12 | 37.33  38.67 | **17.3** |
| **0.30** | 301.29  308.47 | 150.35  154.23 | 100.43  102.82 | 75.32  77.12 | 60.26  61.69 | 50.22  51.41 | 43.04  44.07 | **19.6** |
| **0.40** | 307.44  314.76 | 153.72  157.38 | 102.48  104.92 | 76.86  78.69 | 61.49  62.95 | 51.24  52.46 | 43.92  44.97 | **15** |
| **0.40** | 352.53  360.93 | 176.26  180.46 | 117.5  120.31 | 88.13  90.23 | 70.50  72.19 | 58.76  60.15 | 50.36  51.56 | **17.3** |
| **0.40** | 401.72  411.29 | 200.86  205.64 | 133.91  137.09 | 100.43  102.82 | 80.34  82.26 | 66.95  68.55 | 57.39  58.76 | **19.6** |

Table A. The orthotropic elastic moduli ($E_{\mathrm{circumferential}}, E_{\mathrm{longitudinal}}=E_{\mathrm{radial}}$) for varying cell wall thickness, turgor pressure and pollen tube diameters calculated from the logarithmic strain model.


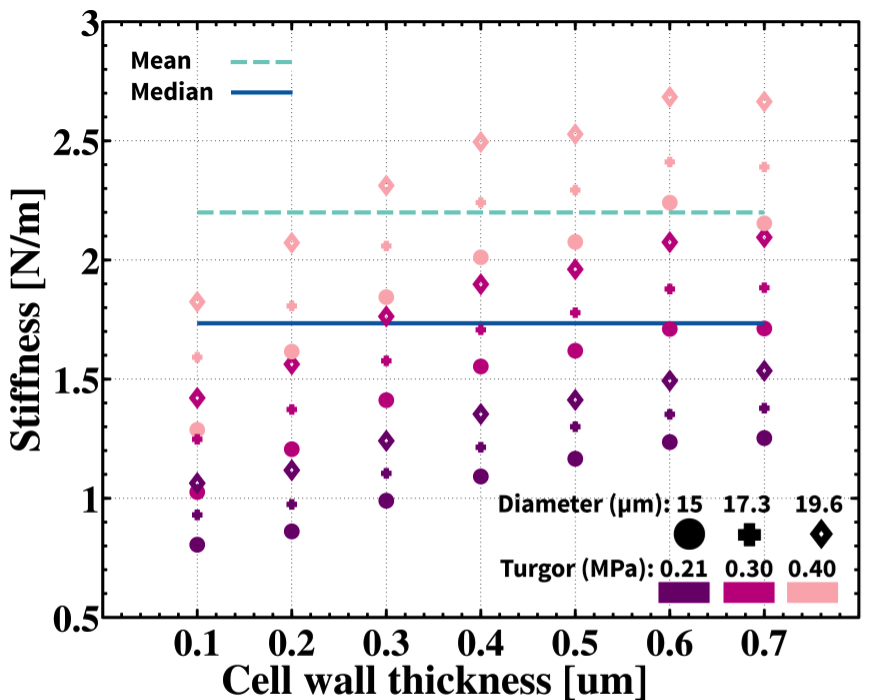


Fig F. Variation of the apparent stiffness of the cell wall of *Lilium longiflorum* pollen tube with varying cell wall thickness, turgor pressure, tube diameter as calculated by FEM analysis. The corresponding orthotropic elastic moduli can be found in Table S3. The experimentally measured mean and median of the loading force-indentation curves are marked on the graph. We note that several different combinations of the input parameters yield similar values of apparent stiffness.

**References:**

1. Vogler H, Draeger C, Weber A, Felekis D, Eichenberger C, Routier-Kierzkowska AL, et al. The pollen tube: A soft shell with a hard core. Plant J. 2013;73: 617–627. doi:10.1111/tpj.12061

2. Weber A, Braybrook S, Huflejt M, Mosca G, Routier-Kierzkowska A-L, Smith RS. Measuring the mechanical properties of plant cells by combining micro-indentation with osmotic treatments. J Exp Bot. 2015;66: 3229–3241. doi:10.1093/jxb/erv135

3. Hearn EJ. Mechanics of Materials, Vol 1. Elsevier. Elsevier; 1997.

4. Wilsen KL, Hepler PK. Sperm Delivery in Flowering Plants: The Control of Pollen Tube Growth. Bioscience. 2007;57: 835. doi:10.1641/B571006

5. McKenna ST, Kunkel JG, Bosch M, Rounds CM, Vidali L, Winship LJ, et al. Exocytosis precedes and predicts the increase in growth in oscillating pollen tubes. Plant Cell. 2009;21: 3026–3040. doi:10.1105/tpc.109.069260

6. Anderson JR, Barnes WS, Bedinger P. 2,6-Dichlorobenzonitrile, a cellulose biosynthesis inhibitor, affects morphology and structural integrity of petunia and lily pollen tubes. J Plant Physiol. 2002;159: 61–67. doi:10.1078/0176-1617-00651

7. Benkert R, Obermeyer G, Bentrup F-W. The turgor pressure of growing lily pollen tubes. Protoplasma. 1997;198: 1–8. doi:10.1007/BF01282125

8. Pertl H, Pöckl M, Blaschke C, Obermeyer G. Osmoregulation in Lilium pollen grains occurs via modulation of the plasma membrane H+ ATPase activity by 14-3-3 proteins. Plant Physiol. 2010;154: 1921–1928. doi:10.1104/pp.110.165696
